# Impact of mechanical circulatory support and immunomodulation therapy on outcome of patients with fulminant myocarditis: Chinese registry of fulminant myocarditis

**DOI:** 10.1038/s41392-021-00700-6

**Published:** 2021-10-06

**Authors:** Ning Zhou, Yuhua Zhao, Jiangang Jiang, Lan Shen, Junming Li, Jing Wan, Xueping Ma, Jing Zhang, Enrico Ammirati, Dao Wen Wang

**Affiliations:** 1 Division of Cardiology, Department of Internal Medicine, Tongji Hospital, Tongji Medical College, Huazhong University of Science and Technology, and Hubei Key Laboratory of Genetics and Molecular Mechanisms of Cardiological Disorders, Wuhan, China; 2 Division of Cardiology, Dongguan Kanghua Hospital, Dongguan, China; 3 Division of Cardiology, Shanghai Chest Hospital, Shanghai Jiao Tong University, Shanghai, China; 4 Division of Cardiology, The First Hospital of Yichang City, Yichang, China; 5 Division of Cardiology, Zhongnan Hospital of Wuhan University, Wuhan, China; 6 Division of Cardiology, General Hospital of Ningxia Medical University, Yinchuan, China; 7 Division of Cardiology, Henan Provincial People’s Hospital, Zhengzhou, China; 8 “De Gasperis” Cardio Center and Transplant Center, ASST Grande Ospedale Metropolitano Niguarda, Milan, Italy

**Keywords:** Health sciences, Cardiology, Cardiovascular diseases

**Dear Editor**,

Patients presenting with acute myocarditis and sudden hemodynamic instability (termed fulminant myocarditis [FM]) still have a high mortality and need for heart transplantation, up to 28% at 60 days.^1–3^ Recent scientific statements and expert opinion consensus suggests early use of temporary mechanical circulatory supports (t-MCS).^3,4^ Specifically, Chinese scientific statement proposed an extensive use of t-MCS combined with immunoregulatory therapy (IT),^4^ although formal trials are lacking. We present a multicenter, retrospective study to compare the outcome of patients who were treated with t-MCS and IT vs. patients who didn’t receive these treatments. We included patients with the diagnosis of FM based on the presence of viral prodromal signs/symptoms followed by acute onset of severe heart failure (HF) without other relevant differential diagnosis or pre-existing cardiac disorders. Patients who received both t-MCS and IT during hospitalization were classified as t-MCS+IT group. T-MCS used were intra-aortic balloon pulsation (IABP) (median duration 7 days, first to third quartile [Q1–Q3, 4–8]) or venous-arterial extracorporeal membrane oxygenation (VA-ECMO) (median duration 5 days [Q1–Q3, 5–7], or both. IT included methylprednisolone (200–400 mg) or dexamethasone (20–40 mg), qd for 3–5 days of intravenous (IV) and then gradually down titrated and weaned in 7–10 days, and IV immunoglobulin (10–20 g qd for the 3–5 days and then 10 g for another 3–5 days). Patients who didn’t receive both t-MCS and IT were classified as non-t-MCS+IT 3 group. Statistical differences were analyzed using the Mann–Whitney *U* test for continuous variables. Categorical variables were compared using Fisher’s exact test or *χ*^2^ test.

A total of 138 FM patients with a median age of 33 years (Q1–Q3, 26–41) and a male prevalence of 55.1% were included in the study (Fig. 1a, Supplementary Table 1). Among 138 patients, 96 (69.6%) patients received t-MCS+IT during hospitalization (median age 32 years [Q1–Q3, 26–44]; 55.2% men). Forty-two patients (30.4%) were in the non-t-MCS+IT group (median age 33 years [Q1–Q3, 25–42]; 54.8% men), among them 16 patients received only t-MCS, 17 patients received only IT and 9 patients received neither t-MCS nor IT. The two groups of FM patients didn’t differ for cardiovascular risk factors, systolic blood pressure (SBP), heart rate (HR), clinical presentation, laboratory findings, and presence of severe ventricular arrythmias (VAs) except for a higher prevalence of fever in t-MCS+IT group compared with non-t-MCS+IT (74.0% vs. 42.9% respectively, *p* < 0.001).

**Fig. 1:**
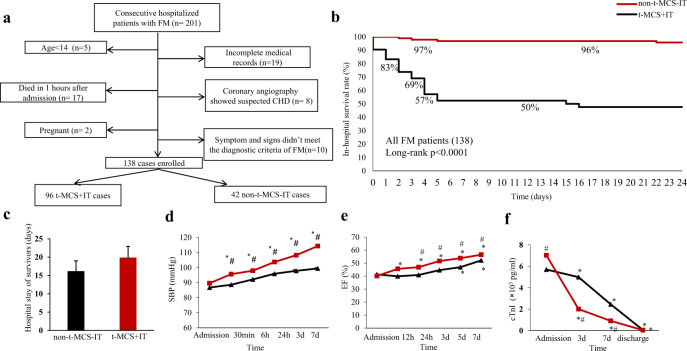
**a** Flow diagram describing the selection of 138 subjects from the overall population diagnosed as FM. **b** The Kaplan–Meier curves of FM patients treated by different methods. **c** The hospital stays length of survived FM patients in both groups. **d** The changes of systolic blood pressure (SBP). *p < 0.05 vs baseline. ^#^*p* < 0.05 vs non-t-MCS+IT group. **e** The changes of ejection fraction of left ventricle. **f** The changes of troponin I of FM patents. *p < 0.05 vs baseline. ^#^*p* < 0.05 vs non-t-MCS+IT group

Overall, in-hospital mortality of FM patients was 18.8% (26 out of 138). We observed 4.2% deaths (4 of 96 patients) in the t-MCS+IT group vs. 52.4% (22 of 42 patients) in the non-t-MCS+IT group (Unadjusted OR 0.03, 95% confidence interval [CI] 0.13–0.92, *p* = 0.001) (Supplementary Table 2). Both t-MCS and IT contributed to the reduction of mortality, however, separate use of t-MCS and IT were associated with a higher mortality compared to combined t-MCS and IT (Supplementary Table 2). Neither heart transplantation nor long-term left ventricular assist device implant was performed. In-hospital mortality risk of FM patients was reduced by 92.7% in t-MCS+IT group (Fig. 1b). Multiorgan failure due to irreversible cardiogenic shock was the cause of all deaths. In the logistic regression model after adjusting for age, gender, and inotrope agents, the use of t-MCS+IT was associated with lower all-cause mortality (adjusted OR, 0.11; 95% CI, 0.09–0.46; *p* = 0.001) vs non-t-MCS+IT group (Supplementary Table 3). Patients with VAs and/or advanced atrioventricular block (AVB) were found to be more beneficial from t-MCS + IT (adjusted OR 0.08, 95% CI 0.01–0.62; adjusted OR 0.32, 95% CI 0.10–0.996, respectively). Among the survivors, t-MCS+IT and non-t-MCS+IT patients had similar hospital stay (Fig. 1c).

Apart from the different use of t-MCS and IT, in the non-t-MCS+IT group there was a significant higher proportion of patients on inotropic agents, whereas invasive ventilation and continuous veno-venous hemodialysis were used only in the t-MCS+IT group (Supplementary Table 4).

After 30 min since institution of t-MCS, we observed a significant improvement in SBP compared to baseline. Patients in the t-MCS+IT showed higher SBP in the first 7 days compared to the non-t-MCS+IT (Fig. 1d). Left ventricular ejection fraction (LVEF) was improved more in the t-MCS+IT group compared with non-MCS+IT group, from a median LVEF of 40% at baseline to 57% in t-MCS+IT survivors (Fig. 1e). Furthermore, troponin I levels dropped more rapidly in the t-MCS+IT group compared with non-t-MCS+IT group (Fig. 1f).

In summary, we observed that the use of t-MCS plus IT was associated with lower risk of death in FM patients. T-MCS allows patients to tide over the crisis, combined IT can wane myocardial inflammation, which together contributed to the striking reduction of in-hospital mortality of FM patients. A study limitation is that no histologic findings confirmed the clinical diagnosis of FM which is recommended as a most important diagnostic criteria of FM,^5^ even if the clinical presentation and the course are typical, thus alternative diagnosis are unlikely. Our findings support the importance of combined application of t-MCS and IT in the treatment of FM, although more confirmative clinical evidence are needed.

## Supplementary information

SUPPLEMENTAL MATERIAL

## Data Availability

The data are available from the corresponding author on reasonable request.
