# Supplementary material for: Impact of mechanical circulatory support and immunomodulation therapy on outcome of patients with fulminant myocarditis: Chinese registry of fulminant myocarditis
Source: Signal Transduct Target Ther. 2021 Oct 6;6:350. doi: 10.1038/s41392-021-00700-6 (PMC8492670; doi:10.1038/s41392-021-00700-6)
Supplement: Supplementary file 1 — SUPPLEMENTAL MATERIAL [file 41392_2021_700_MOESM1_ESM.docx]

Supplementary Materials for

Impact of mechanical circulatory support and immunomodulation therapy on outcome of patients with fulminant myocarditis: Chinese registry of fulminant myocarditis

Ning Zhou^1^, Yuhua Zhao^2^, Jiangang Jiang^1^, Lan Shen^3^, Junming Li^4^, Jing Wan^5^,

Xueping Ma^6^, Jing Zhang^7#^, Enrico Ammirati^8#^, Dao Wen Wang^1#^

^#^Corresponding authors:

Dao Wen Wang, MD, PhD

Department of Internal Medicine, Tongji Hospital, Tongji Medical College, Huazhong University of Science & Technology, 1095 Jiefang Ave., Wuhan 430030, PRC, Tel. & Fax: 86-27-8366-3280. dwwang@tjh.tjmu.edu.cn

Enrico Ammirati, MD, PhD

"De Gasperis" Cardio Center and Transplant Center, ASST Grande Ospedale Metropolitano Niguarda, Piazza Ospedale Maggiore 3, 20162 Milan, Italy enrico.ammirati@ospedaleniguarda.it

Jing Zhang, MD

Division of Cardiology, Fuwai Central China Cardiovascular Hospital, 1 Fuwai Ave. Zhengzhou 450003, China. zhangjingxnk@qq.com

**This PDF file includes:**

Tables S1 to S4

Table S1. Baseline characteristics of the patients with fulminant myocarditis

| Variables | All (n=138) | t-MCS+IT (n=96) | Non-t-MCS-IT (n=42) | *P* value |
| --- | --- | --- | --- | --- |
| Age, yr, median (Q1-Q3) | 33 (26-44) | 32 (25-42) | 33 (29-46) | 0.133 |
| Female, n.(%) | 62 (44.9) | 43 (44.8) | 19 (45.2) | 0.961 |
| Hypertension, n.(%) | 18 (13) | 13 (13.5) | 5 (14.2) | 0.793 |
| DM, n. (%) | 13 (9.4) | 9 (9.4) | 4 (9.5) | 0.978 |
| SBP,mmHg, median (Q1-Q3) | 88 (80-97) | 87 (80-100) | 88 (80-91) | 0.74 |
| DBP,mmHg, median (Q1-Q3) | 56 (50-64) | 60 (50-66) | 56 (50-62) | 0.606 |
| Heart rate (beats/min) | 104 (76-130) | 104 (76-131) | 105 (78-120) | 0.646 |
| Fever (%) | 89 (64.5) | 71 (74.0) | 18 (42.9) | <0.001* |
| Body temperature (°C) | 36.6 (36.3-37.7) | 36.6 (36.4-37.7) | 36.5 (36.3-37.8) | 0.894 |
| Diarrhea (%) | 14 (10.1) | 12 (12.5) | 2 (4.8) | 0.227 |
| Unconsciousness (%) | 25 (18.1) | 20 (20.8) | 5 (11.9) | 0.21 |
| Chest pain (%) | 52 (37.7) | 40 (41.7) | 12 (28.6) | 0.144 |
| Chest discomfort (%) | 118 (85.5) | 82 (85.4) | 36 (85.7) | 0.964 |
| ECG |  |  |  |  |
| Conduction block (%) | 43 (31.1) | 33 (34.4) | 10 (23.8) | 0.218 |
| VT/VF (%) | 32 (23.7) | 23 (24) | 9 (21.4) | 0.746 |
| Pericardial effusion (%) | 12 (8.7) | 8 (8.3) | 4 (9.5) | 0.097 |
| Troponin I (pg/mL) | 2000 (98-7816) | 2000 (122-8920) | 850 (41-2972) | 0.21 |
| NT-proBNP (pg/mL) | 4826 (1146-12234) | 4011 (995-10586) | 10862 (3234-15126) | 0.081 |
| ALT (U/L) | 150 (49-238) | 136 (49-240) | 174 (81-234) | 0.36 |
| Creatinine (mmol/L) | 87 (64-141) | 91 (66-149) | 78 (63-119) | 0.344 |

ECG, electrocardiography; DBP, diastolic blood pressure, DM, diabetes mellitus; IT immunomodulatory therapy (corticosteroids, immunoglobin); Q1-Q3, first to third quartile; SBP, systolic blood pressure; t-MCS, indicates temporary mechanical circulatory support; VT, ventricular tachycardia; VF, ventricular fibrillation.

Table S2. Crude in-hospital outcomes according to t-MCS+IT.

| Outcome | Cumulative incidence | | | Unadjusted OR* (95% CI) | P value |
| --- | --- | --- | --- | --- | --- |
|  | Overall | t-MCS+IT | Non-t-MCS+IT |  |  |
| In-hospital mortality | 26/138 (18.8%) | 4/96 (4.2%) | 22/42 (52.4%) | 0.03 (0.13-0.92) | p=0.001 |

t-MCS IT none

6/16 10/17 6/9

(37.5%) (58.8) (66.7%)

IT immunomodulatory therapy (corticosteroids and intravenous immunoglobulins); t-MCS, indicates temporary mechanical circulatory support; the combined use of t-MCS and IT was associated with lower all-cause mortality (adjusted OR, 0.03; 95% CI, 0.01-0.92; *p*=0.001) vs Non- t-MCS+IT group. The use of t-MCS was associated with lower all-cause mortality (adjusted OR, 0.16; 95% CI, 0.06-0.41; *p*=0.004) vs none group, whereas higher all-cause mortality (adjusted OR, 0.07; 95% CI, 0.02-0.67; *p*=0.003) vs t-MCS+IT group. the all-cause mortality of IT group was lower compared to none group (adjusted OR, 0.28; 95% CI, 0.05-0.54; *p*=0.01) but significantly higher than t-MCS group (adjusted OR, 0.11; 95% CI, 0.05-0.56; *p*=0.001) and t-MCS+IT group (adjusted OR, 0.02; 95% CI, 0.03-0.51; *p*=0.001).

Table S3. Adjusted in-hospital mortality rate compared by t-MCS+IT versus non-t-MCS-IT, before and after propensity score weighting, respectively.

| Outcomes | Unweighted | | Weighted | |
| --- | --- | --- | --- | --- |
|  | Adjusted OR (95% CI) | P value | Adjusted OR (95% CI) | P value |
| In-hospital mortality | 0.11 (0.09-0.46) | *p*=0.001 | 0.18 (0.10-0.30) | *p*=0001 |
| VT/VF and/or advanced AVB | 0.08 (0.01-0. 62) | *p*=0.016 | 0.32 (0.01-0.996) | *p*=0.002 |
| Non VT/VF and/or advanced AVB | 0.31 (0.04-0.58) | *p*=0.039 | 0.24 (0.02-0.28) | *p*=0.095 |

IT immunomodulatory therapy (corticosteroids and intravenous immunoglobulins); t-MCS, indicates temporary mechanical circulatory support; VT, ventricular tachycardia; AVB, atrioventricular block;

Table S4. Treatment of the patients with fulminant myocarditis.

| Variables n (%) | t-MCS+IT  (n=96) | Non-t-MCS-IT (n=42) | P |
| --- | --- | --- | --- |
| methylprednisolone | 85 (88.5) | 12 (28.6) | 0.001 |
| IVIG | 69 (71.9) | 5 (11.9) | 0.001 |
| dexamethasone | 55 (57.3) | 7 (16.7) | 0.001 |
| IVIG + methylprednisolone | 60 (62.5) | 4 (9.5) | 0.001 |
| IVIG + dexamethasone | 41 (42.7) | 3 (7.1) | 0.001 |
| dopamine | 36 (37.5) | 34 (80.9) | 0.001 |
| norepinephrine | 31 (32.3) | 33 (78.6) | 0.001 |
| ACEi/ARB | 10 (10.4) | 14 (33.3) | 0.087 |
| β-Blocker | 19 (19.8) | 15 (35.7) | 0.061 |
| coenzyme Q10 | 82 (85.4) | 23 (54.7) | 0.041 |
| IABP | 81 (84.3) | 11(26.2) | 0.001 |
| VA-ECMO | 46 (47.9) | 5 (11.9) | 0.003 |
| IABP+VA-ECMO | 35 (36.5) | 2 (4.8) | 0.002 |
| Invasive ventilation | 31 (32.3) | 0 |  |
| CVVH, n(%) | 29 (30.2) | 0 |  |

ACEi, angiotension converting enzyme inhibitor. ARB, Angiotensin II receptor 1

blocker; CVVH, continuous veno-venous hemodialysis; IABP, intra-aortic balloon

pulsation; IT immunomodulatory therapy (corticosteroids and intravenous

immunoglobulins [IVIG]); t-MCS, indicates temporary mechanical circulatory

support; VA-ECMO, venous-arterial extracorporeal membrane oxygenator.
